# More Concerns for Farmers: Neurologic Effects of Chronic Pesticide Exposure

**Published:** 2005-07

**Authors:** Julia R. Barrett

Although there is considerable evidence that pesticides are neurotoxic, most research has focused on the short- and long-term consequences of acute high-level exposure such as that seen during industrial accidents or food contamination. To date, little has been known about the effects of chronic moderate exposure such as that experienced by farmers and other workers who regularly use agricultural pesticides. Now, a recent analysis of data collected in the Agricultural Health Study (AHS) links chronic moderate pesticide exposure to neurologic symptoms affecting both the central and peripheral nervous systems **[*EHP* 113:877–882]**. According to the research team, increases in such symptoms may be an early indicator of impaired neurological function.

The AHS, an ongoing study sponsored by the NIEHS, the National Cancer Institute, and the U.S. Environmental Protection Agency, furnished a rich data source for the researchers to investigate possible links. Between 1993 and 1997, approximately 20,000 private pesticide applicators (primarily farmers) in Iowa and North Carolina enrolled in the AHS and completed two questionnaires on demographic characteristics, lifestyle, medical history (including neurologic symptoms), and pesticide use. The current analysis focused on 18,782 of these individuals, white men aged 18–75 years who provided complete symptom information.

The 23 symptoms in the analysis included headache, dizziness, depression, limb weakness, poor balance, difficulty concentrating, and vision difficulties. In addition to the symptom information, participants detailed how long and how frequently they used any of 50 pesticides, including insecticides, herbicides, fungicides, and fumigants. They also indicated whether they had ever experienced pesticide poisoning or high-exposure incidents such as accidental skin contact with a large amount of pesticide.

To define cumulative exposure, the researchers calculated lifetime days of use from the number of years and the number of days per year that the applicators had used each pesticide. The team considered two measures of symptoms: the absolute number and the presence of 10 or more. To control for confounding by pesticide poisoning or high-exposure incidents, the researchers conducted analyses with and without those data from affected individuals. They also considered potential effects from pesticide use within the past year.

For pesticides overall, applicators with the most (more than 500) cumulative lifetime days of pesticide use reported more symptoms than those with the fewest lifetime days of use. The relationship between cumulative exposure and symptoms was strongest with insecticides; applicants with the most lifetime days of use were 2.5 times more likely to have 10 or more symptoms as applicators who had never used insecticides. Within the insecticide category, relationships with symptoms were strongest for organophosphates and organochlorines. Neither recent use nor a history of poisoning or high-exposure incident affected the results.

The results of this study extend previous research demonstrating a link between chronic moderate pesticide exposure and a range of cognitive, sensory, and motor symptoms. The AHS is unusually robust due to its large size and its wealth of detailed exposure information. The results of this analysis provide substantial evidence that neurologic symptoms may be increased by even moderate insecticide exposure, and that cumulative exposure may be as important as recent exposure, although more work is needed to understand the pathology underlying the reported symptoms.

## Figures and Tables

**Figure:**
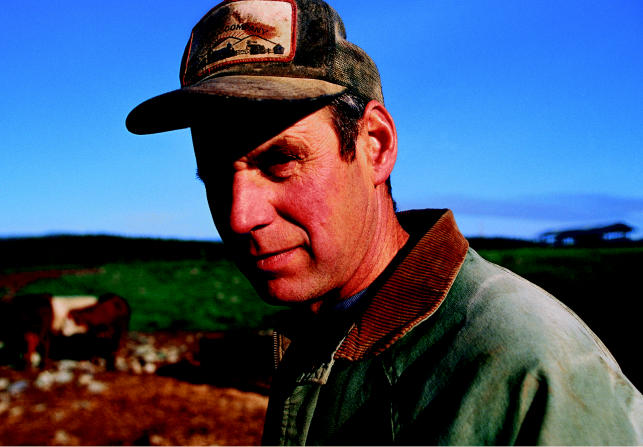
**Farm field fallout.** A recent analysis shows that even moderate chronic pesticide use can result in neurologic symptoms among farmers and other applicators.

